# Study on the multifunctional spatial-temporal evolution and coupling coordination of cultivated land: A case study of Hebei Province, China

**DOI:** 10.1371/journal.pone.0306110

**Published:** 2024-07-01

**Authors:** Shijie Liu, Yapeng Zhou, Yuepu Qi, Yaheng Chen, Wei Liu, Hao Xu, Shutao Wang

**Affiliations:** 1 College of Land and Resources, Hebei Agricultural University, Baoding, China; 2 Shijiazhuang Shibro Land Environmental Technology Service Co., Ltd., Shijiazhuang, China; 3 The Key Laboratory of Agricultural Ecological Environment of Hebei Province, Baoding, China; 4 College of Chemistry and Materials Science, Hebei University, Baoding, China; Universidade Federal de Uberlandia, BRAZIL

## Abstract

The rational use of cultivated land can guarantee food security and thus is highly important for ensuring social stability, economic development and national security. The current study investigated the multifunctional temporal and spatial variation characteristics of cultivated land and explored the spatial and temporal characteristics of the multifunction and coupling coordination degrees of cultivated land throughout Hebei Province. Based on the administrative division data, statistical yearbook data and land use status data of the impacted areas, a multifunctional evaluation index system of cultivated land was established. The CRITIC weight method and entropy weight method were used to determine the weight of the index, the comprehensive index model was used to determine the production, social security, ecology and landscape functions of cultivated land of Hebei Province in different periods, the coupling coordination model was used to explore the multifunctional coupling coordination degree of cultivated land in each county, and spatial autocorrelation analysis was performed to determine the correlation of the multifunctional coupling coordination degrees. From 2000 to 2020, the production, social security and landscape function of cultivated land in Hebei Province trended upward; the ecological function trended slightly downward. The multifunctional coupling coordination degree of cultivated land in Hebei Province trended significantly upward and changed from limited coordination to intermediate coordination. Furthermore, it exhibited strong agglomeration and a significant positive spatial correlation, forming a ’V’-type change rule of first decreasing and then increasing. Hebei Province exhibited remarkable spatial and temporal characteristics of the multifunction and coupling coordination degrees of cultivated land. Regions could thus customize different cultivated land functions to maximize the benefits of cultivated land use. The findings of this study may provide a scientific basis and theoretical support for sustainably using and managing cultivated land resources in areas with similar human geographical environments.

## Introduction

Cultivated land, as an indispensable component of land, matters both the present and future of human beings [1]. In the context of urbanization and rapid economic development, urban-rural spatial structure, industrial structure, and population structure are all experiencing a period of rapid transformation. Disorderly expansion of construction land and irrational cultivated land utilization have led to problems such as disintensification, non-agriculturalization, marginalization, and fragmentation of cultivated land functions, posing a threat to food security and sustainable cultivated land utilization [2, 3]. Apart from demands of urban construction and development, people’s insufficient understanding of the functions of arable land also plays an important role in the ineffective protection of arable land [4]. This incomplete understanding has caused a high degree of asymmetry between the main body and beneficiaries of cultivated land protection, resulting in cultivated land non-agriculturalization. In addition, chemical fertilizers, pesticides and plastic film are widely used on cultivated land; their use leads to a decline in cultivated land quality, a large amount of cultivated land waste, cultivated land pollution and other problems [5]. Therefore, multifunctionality of cultivated land has become a hot topic of cultivated land protection nowadays.

The multifunctionality of cultivated land means that in addition to its production functions, such as food production, cultivated land also has functions such as social security, ecological protection, and landscape culture [6]. Food is a paramount necessity, and the production function of cultivated land provides the material basis for ensuring this most basic survival need [7]. Cultivated land is the foundation of all material production. Due to the limited quantity of cultivated land and the amount of labor it requires, cultivated land is considered a type of property that can ensure survival and provide livelihoods. Cultivated land has naturally become an important aspect of human society, and the social security function of cultivated land has emerged [8]. Land is the carrier of ecological civilization and the key guarantee for ecological civilization construction, and thus its ecological construction should be emphasized [9]. With the acceleration of urbanization, people’s pursuit of spiritual life is increasing, which promotes the demand for cultural landscapes and accelerates the realization of the landscape function of farmland.

Generally, research on the multiple functions of cultivated land has been performed at three scales: macroscale, mesoscale and microscale. At the macroscale, the province is the research unit [10]; at the mesoscale, the city is the research unit [11]; and at the microscale, the county is the research unit [12]. In terms of methods, a variety of approaches have been used for the multifunctional application of cultivated land, such as multiple factor comprehensive analysis, spatial analysis and a gray correlation model in Niu et al. study [13], the entropy method and Spearman rank correlation in Yang et al. study [14], root mean square difference in Yin et al. study [15], and geospatial models and statistical analysis in Fan et al. study [6]. In determining the weight values of indicators, scholars typically choose the single weight weighting method [16], which has certain limitations. The application of combined weighting evaluation models in multifunctional evaluation of cultivated land is relatively limited. For instance, criteria importance though intercriteria correlation (CRITIC) is an objective weighting method that is based mainly on the comparative strength within the evaluation indicators and the degree of conflict between the indicators. It can comprehensively determine the objective weights of the indicators. Additionally, CRITIC not only considers the variability of indicators but also accounts for the correlation between indicators and thus is completely based on the objective attributes of the data itself for scientific evaluation. However, the CRITIC method has its limitations, as it cannot display and measure the degree of dispersion between various evaluation indicators. In contrast, the entropy weight method has good suitability for measuring the degree of dispersion of indicators [17]. In this situation, the comprehensive application of the CRITIC method and entropy weight method may more objectively reflect the weight of the evaluation indicators and increase the accuracy of the evaluation results. In terms of the interactions among the different functions of cultivated land, most scholars have focused on the separate evaluation of the functions [18]. Although an increasing number of scholars have gradually turned their attention to the trade-offs and collaborative relationships between various functions [6, 14–15, 19], they still mostly limit their studies to the analysis of pairwise relationships between various functions; studies regarding the quantitative expression of the coupling relationships between multifunctional systems in cultivated land remain relatively limited.

Therefore, this paper selected Hebei Province as an example to study the temporal and spatial evolution and coupling coordination of multifunctional cultivated land in each county. The CRITIC weight method and entropy weight method were used to determine the weight of each index. The comprehensive index model was used to evaluate the production, social security, ecology and landscape functions of cultivated land in different periods in Hebei Province. The coupling coordination degree and spatial autocorrelation model were used to explore the spatial and temporal characteristics of the multifunctional coupling coordination degree of cultivated land in each county and reveal its spatial distribution law.

## Materials and methods

### Study area

Hebei Province is surrounded by the capital Beijing, at 113°27 x-119°50′E, 36°05′-42°40′N, with a provincial area of 1.89×10^7^ hm^2^. In 2020, the resident population of Hebei Province was 7.46×10^7^. Hebei Province has a temperate continental monsoon climate. The distribution of precipitation is characterized by more precipitation in the southeast and less precipitation in the northwest. The terrain is high in the northwest and low in the southeast. It is an important grain-producing area in North China [20].

**Fig 1:**
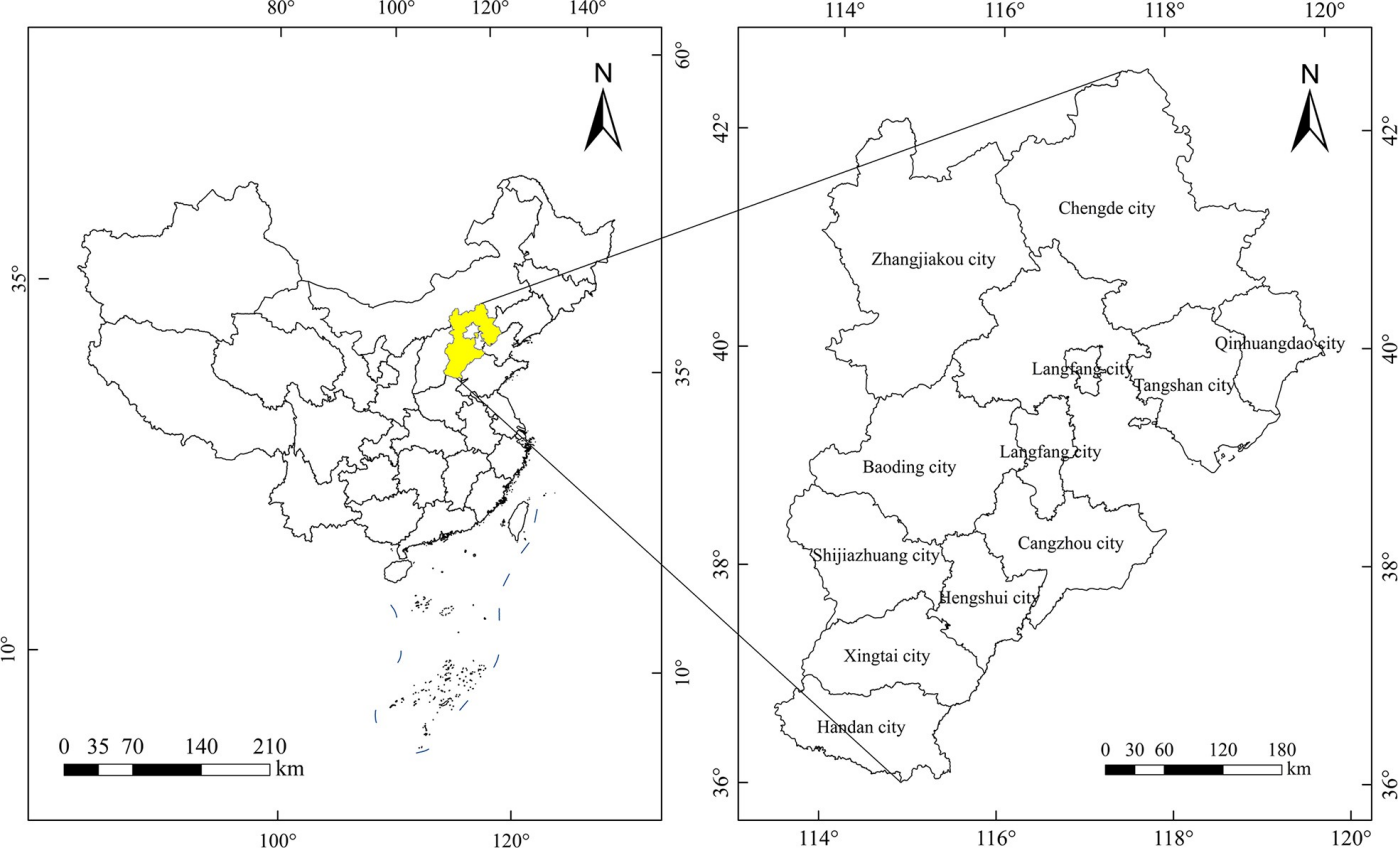
The regional district and administrative division maps of Hebei Province (drawn with ArcGIS).

Hebei Province has two major mountain ranges: the Yanshan Mountains and the Taihang Mountains. The Yanshan Mountains are located in the northern part of the province, which spans Zhangjiakou city, Chengde city, and Qinhuangdao city. The Taihang Mountains are located in the western part of the province, running across Handan city, Xingtai city, Shijiazhuang city, and Baoding city. The regional district and administrative division maps of Hebei Province are shown in Fig 1.

### Data sources

The socioeconomic statistics of Hebei Province were obtained from the ’Hebei Statistical Yearbook’ and ’Hebei Rural Statistical Yearbook’ for 2001, 2011 and 2021, and some data were obtained from the statistical yearbooks of various cities. The vector data for the administrative division of Hebei Province and the land use status data at a 1 km resolution (2000, 2010, 2020) were obtained from the Resource and Environmental Science and Data Center of the Chinese Academy of Sciences (https://www.resdc.cn/). Considering the impact of incomplete data in some districts and counties on functional evaluation results, the deletion method was used in this study to address the missing data problem and 135 counties and districts in the study area were identified as the basic research units. These counties and districts had relatively complete and reliable index data and could represent different types and characteristics of cultivated land functions.

### Construction of a multifunctional index system of cultivated land

In this paper, the function of cultivated land was divided into four categories, namely, production, social security, ecology and landscape, according to both the literature [6] and the actual developmental stages of the concept of the multifunctionality of cultivated land in China. According to scientific, systematic, operable and objective principles [21], combined with the actual situation of cultivated land in Hebei Province, a multifunctional evaluation index system of cultivated land was established, and 19 indices were finally determined, including grain crop yield, grain self-sufficiency rate and other indicators (Table 1).

**Table 1:** Multifunctional evaluation index system for farmland.

Function type	Indicator layer	Method of calculation	Property	Index weight	Functional weight
Production function	Grain crop yield A1	Grain yield/grain sown area	+	0.0412	0.3074
	Cultivated land reclamation rate A2	Cultivated land area/total land area	+	0.0633	
	Farmland area per person A3	Cultivated land area/household population	+	0.0776	
	Average agricultural output value of cultivated land A4	Total agricultural output value/cultivated land area	+	0.0878	
	Multiple cropping coefficient A5	Total sown area of crops/total cultivated land area	+	0.0375	
Social security function	Grain self-sufficiency rate B1	Grain yield/(resident population × 400 kg/per)	+	0.0444	0.3053
	Contribution rate of agriculture to GDP B2	Gross agricultural output/regional GDP	+	0.0728	
	Rural net income per capita	From the Statistical Yearbook	+	0.1131	
	Agricultural mechanization level B4	Total power of agricultural machinery/cultivated land area	-	0.0248	
	The number of cultivated land Carrying labor force B5	Number of regional agricultural employees/cultivated land area	+	0.0502	
Ecological function	Fertilizer application intensity C1	Fertilizer application rate/cultivated land area	-	0.0238	0.1449
	Pesticide application intensity C2	Pesticide application rate/cultivated land area	-	0.0186	
	Mulch film use intensity C3	Plastic film usage/cultivated land area	-	0.0345	
	Farmland ecosystem diversity index C4	-∑*b*_*i*_ ln *b*_*i*_, *b*_*i*_ is The ratio of the sown area of each crop to the total sown area of the crop.	+	0.0429	
	Per capita ecological carrying capacity of cultivated land C5	Per capita cultivated land resource endowment × cultivated land yield factor × cultivated land equilibrium factor	-	0.0251	
Landscape function	Landscape demand level D1	Per capita GDP (RMB/per)	+	0.0859	0.2424
	Field regularity D2	Calculated using Fragstats software	+	0.0898	
	Landscape fragmentation D3	Calculated using Fragstats software	-	0.0241	
	Landscape aggregation degree D4	Calculated using Fragstats software	+	0.0426	

The production function of cultivated land provides various crops for humans to meet their basic needs [22], and three perspectives were taken into consideration in this study i.e., grain production level, cultivated land development and utilization status, and human and material resource input level [23]. The grain yield per unit area refers to the overall production capacity of grain yield per unit area, which is positively correlated with the production capacity of cultivated land. The multiple cropping index is an indicator used to show the status of farming in various regions, and it can further describe the level of cultivated land output capacity. The reclamation rate of cultivated land can effectively reflect the development and utilization of cultivated land. The per capita cultivated land area and the agricultural output value per unit area of cultivated land show the utilization of cultivated land resources and the input level of agricultural production factors per unit area.

The social security function has important social and economic value for farmers [24], where, three aspects are considered, i.e., food security, economic security and employment security. The food self-sufficiency rate indicates the food security level of cultivated land. The contribution rate of agriculture to GDP and the per capita net income of rural areas reflect the important role of cultivated land in national economic development and rural residents’ economic security. The total power of agricultural machinery per unit area can be used to measure the employment carrying capacity of cultivated land. The employment security capacity of cultivated land for regional agricultural employees can also be reflected by the number of laborers carried by cultivated land.

Ecological function reflects the ability of cultivated land to maintain biodiversity and the ecological environment. In addition to the attributes of cultivated land resources, ecological function is affected by human activities such as pesticides and fertilizers [25]. For the farmland ecosystem diversity index, six kinds of food crops and economic crops are selected and the calculation method was based on -∑*b*_*i*_ ln *b*_*i*_ [26], where *b*_*i*_ is the ratio of each crop to the sown area of crops. The per capita cultivated land ecological carrying capacity is used to characterize the level of cultivated land ecosystem carrying capacity in a region. The per capita cultivated land ecological carrying capacity is determined by three factors: per capita cultivated land resource endowment, cultivated land yield factor and cultivated land balance factor. Only the impact of cultivated land is considered, so the cultivated land equilibrium factor is set to 1, and the cultivated land yield factor is the ratio of the grain yield per unit area in the study area to the national grain yield per unit area in the year.

The landscape function of cultivated land reflects the complexity of nature and humanity and provides aesthetic value for human beings [27]. Considering the aesthetic needs of the human landscape and the landscape that cultivated land can provide for humans, the demand side is expressed by per capita GDP. It is generally believed that the greater the income level of residents is, the greater the demand for landscape culture. In terms of cultivated land for human supply, clustered and large-scale cultivated land has more aesthetic ornamental value; this land is characterized by field regularity (e.g., landscape shape index, LSI), landscape fragmentation (e.g., patch density, PD) and landscape aggregation (AI). The LSI, PD and AI are all landscape pattern indices, which are first processed by ArcGIS 10.7 software and then imported into Fragstats software to obtain the index values.

### Calculation method

#### Standardization of indicators

To compare the various indicators, the range method [28] was used to convert the original data into dimensionless data. The standardized formulas for the positive and negative indicators are as follows [28]:

$$Positive\;indices:\;X_{ij}=(x_{ij}-x_{j\min})/(x_{j\max}-x_{i\min})$$ (1)

$$Negative\;indicators:\;X_{ij}=(x_{j\max}-x_{ij})/(x_{j\max}-x_{j\min})$$ (2)

where *X*_*ij*_ is the standardized value, *x*_*ij*_ is the actual value of the *j*th index of county *i*, and *x*_*jmax*_ and *x*_*jmin*_ are the maximum and minimum values of the *j*th index, respectively.

#### Weight determination

The index weight of the CRITIC method was calculated according to the internal contrast strength and conflict of evaluation indices; however, the CRITIC method cannot measure the dispersion of each index. The entropy weight method is based on the dispersion of each index to calculate the index weight [29]. Therefore, this study selected the CRITIC method and the entropy weight method to calculate the index weight.

According to the CRITIC method used to calculate the weight, the following formulas are used [29]:

$${\bar{X}}_{j}=\frac{1}{m}\sum_{i=1}^{m}X_{ij}$$ (3)

$$S_{j}=\sqrt{\frac{\sum_{i=1}^{m}\left(X_{ij}-{\bar{X}}_{j}\right)}{m-1}}$$ (4)

$$R_{j}=\sum_{j=1}^{n}\left(1-r_{ij}\right)$$ (5)

$$C_{j}=S_{j}\cdot R_{j}$$ (6)

$$W_{1}=\frac{C_{j}}{\sum_{j=1}^{n}C_{j}}$$ (7)

where ${\bar{X}}_{j}$ is the average value of the index, *S*_*j*_ is the standard deviation of the index, *R*_*j*_ is the conflict of the index, *r*_*ij*_ is the coefficient of variation of the index, *C*_*j*_ is the information content of the index, and *W*_*1*_ is the weight of the *j*th index.

The following formulas were used to calculate the weights via the entropy weight method [14]:

$$Y_{ij}=\frac{X_{ij}}{\sum_{i=1}^{m}X_{ij}}$$ (8)

$$e_{j}=-k\sum_{i=1}^{m}\left(Y_{ij}\cdot \ln Y_{ij}\right)$$ (9)

$$d_{j}=1-e_{j}$$ (10)

$$W_{2}=\frac{d_{j}}{\sum_{j=1}^{n}d_{j}}$$ (11)

where *Y*_*ij*_ is the proportion of the *j*th index of county *i*, *e*_*j*_ is the index information entropy, *d*_*j*_ is the information utility value, and *W*_*2*_ is the index weight of the *j*th index. *k = 1/lnm*, where *m* is the total number of evaluation units and *n* is the number of indicators.

The combined weight of the *j*th index under the combination of the two methods was calculated as follows, taking *β* = 0.5, indicating that the importance of the two methods is the same [30]:

$$W_{j}=\beta W_{1}+(1-\beta )W_{2}$$ (12)

#### Index weight and cultivated land subfunction calculation

The standardized data was combined with the weight value of each index, and the comprehensive index model was used to calculate the subfunction value of cultivated land in each county and district of Hebei Province, with the formula as follows [31]:

$$U=\sum_{j=1}^{\theta }\left(X_{ij}\cdot W_{j}\right)$$ (13)

where *U* is the subfunction index of cultivated land and *θ* is the number of subfunction evaluation indices. According to the actual situation of Hebei Province and previous studies, the equidistant method was used in this study to divide the index into five levels, corresponding to five functional levels, as shown in Table 2.

**Table 2:** Classification criteria of cultivated land function grades and types.

Functional index score	Function grade
[1, 0.8]	Strong
(0.8, 0.6]	Relatively strong
(0.6, 0.4]	Medium
(0.4, 0.2]	Relatively weak
(0.2, 0]	Weak

### Analysis of the multifunctional temporal and spatial variation characteristics of cultivated land

Based on the multifunctional evaluation index system and calculation equations of the functional indices mentioned above, the multifunctional variation characteristics of cultivated land were analyzed both temporally and spatially.

In the temporal dimension, regression analysis [31], which is a widely used statistical method for describing the relationship of a dependent variable with one or several independent variables with the variables presented numerically rather than other presentation forms, was performed in this study. In actual operations, regression analysis is able to determine the quantitative relationships between variables. In this study, a univariate linear regression model used the following equation [32]:

y=kx+a (14)

$$k=\frac{\sum x_{i}y_{i}-\bar{x}\sum y_{i}}{\sum x_{i}^{2}-\bar{x}\sum x_{i}}$$ (15)

$$a=\bar{y}-k\bar{x}$$ (16)

where *x* is the independent variable, *y* is the dependent variable, *α* is the constant, *k* is the regression coefficient, *x*_*i*_ and *y*_*i*_ are the values of the independent and dependent variables of the *i*th sample, respectively, and $\bar{x}$ and $\bar{y}$ are the averages of the independent and dependent variables of the *i*th sample, respectively.

A regression test was performed on the univariate linear regression model as follows [33]:

$$R^{2}=ESS/TSS$$ (17)

$$ESS=\sum {\left(y_{i}^{'}-\bar{y}\right)}^{2}$$ (18)

$$TSS=\sum {\left(y_{i}-\bar{y}\right)}^{2}$$ (19)

$$R^{2}=\frac{\sum {\left(y_{i}^{'}-\bar{y}\right)}^{2}}{\sum {\left(y_{i}-\bar{y}\right)}^{2}}=1-\frac{\sum {\left(y_{i}-y_{i}^{'}\right)}^{2}}{\sum {\left(y_{i}-\bar{y}\right)}^{2}}$$ (20)

where *R*^*2*^ is the coefficient of determination, *ESS* is the explained sum of squares, *TSS* is the total sum of squares, and $y_{i}^{'}$ is the predicted value of the *i*th sample. The coefficient of determination *R*^*2*^ reflects the interpretable proportion of the independent variable to the dependent variable, with a value ranging from 0 to 1. The larger the value is, the greater the interpretable proportion and the greater the degree of fit.

In the spatial dimension, the functional index of cultivated land and its degree in each county were introduced as attribute data into the attribute table of the administrative division layer of Hebei Province. Subsequently, the mapping function of ArcGIS software was used to present and analyze the spatial distribution characteristics of various functions.

#### Calculation of the multifunctional coupling coordination degree of cultivated land

Coupling coordination refers to the evolutionary process from disorder to order between systems or among various elements within a system, with their interrelationships ranging from simple to complex and from low to high. According to system theory [13], there is an internal coupling relationship between the subfunctions in the complex system of cultivated land. The multifunctional coupling coordination degree of cultivated land [6] refers to the degree of benign coupling in the interaction relationships among various functions, which reflects not only the degree of interaction among various functions but also the degree of coordination among them. The degree of coupling coordination among various functions has a direct impact on the development of the research areas. A high degree of coordination can promote development, whereas a low degree can hinder the sustainable development of the related area. In this study, the coupling coordination degree model [34] was used to analyze the coupling coordination mechanism between cultivated land functions. The formulas are as follows [34]:

$$C=\sqrt[{4}]{\frac{U_{1}U_{2}U_{3}U_{4}}{{\left(\frac{U_{1}+U_{2}+U_{3}+U_{4}}{4}\right)}^{4}}}$$ (21)

$$T=W_{1}U_{1}+W_{2}U_{2}+W_{3}U_{3}+W_{4}U_{4}$$ (22)

$$W_{1}+W_{2}+W_{3}+W_{4}=1$$ (23)

$$D=\sqrt{C\cdot T}$$ (24)

where *C* is the coupling degree; *U*_*1*_, *U*_*2*_, *U*_*3*_ and *U*_*4*_ are the indices of cultivated land production, social security, ecology and landscape function, respectively; *T* is the multifunctional comprehensive index of cultivated land; *W*_*1*_, *W*_*2*_, *W*_*3*_ and *W*_*4*_ are the undetermined coefficients of each function, that is, the comprehensive weight value of each function; and *D* is the coupling coordination degree. According to the actual situation of Hebei Province and previous studies [21], the coupling coordination degree was divided into 10 stages and 2 categories (Table 3).

**Table 3:** Classification of the coordination degree of multifunctional coupling of cultivated land.

Coupling coordination degree value	Coupling coordination degree	Coupling coordination degree type	Coupling coordination degree value	Coupling coordination degree	Coupling coordination degree type
(0.0–0.1)	Extreme disorder	Disorders	[0.5–0.6)	Reluctant coordination	Coordination
[0.1–0.2)	Serious disorder		[0.6–0.7)	Primary coordination	
[0.2–0.3)	Moderate disorder		[0.7–0.8)	Intermediate coordination	
[0.3–0.4)	Mild disorder		[0.8–0.9)	Good coordination	
[0.4–0.5)	on the verge of disorder		[0.9–1.0)	High quality coordination	

#### Spatial autocorrelation analysis

Based on the calculation outcomes of the multifunctional coupling coordination degree of cultivated land, the coupling coordination value and coupling coordination degree of cultivated land function in each county were imported as attribute data into the attribute table of the administrative division layer of Hebei Province, and the changing characteristics of coupling coordination were presented using the mapping function of ArcGIS software. To further explore whether there was spatial autocorrelation in the multifunctional coupling coordination of cultivated land in Hebei Province, ArcGIS software and GeoDa software were utilized based on the results obtained above to conduct correlation analysis from the perspectives of both global and local autocorrelation and to confirm whether the coupling coordination degree of different regions was influenced by surrounding cities. In this study, the document that contained the attribute data regarding the coupling coordination value and coupling coordination degree of cultivated land function in each county processed by ArcGIS software was introduced into GeoDa software to construct a spatial weight matrix, and the spatial analysis module of GeoDa was used for global and local autocorrelation analyses.

*(1) Global autocorrelation analysis*. The global autocorrelation revealed the average spatial correlation degree of the whole research unit. This study referred to the similarity of the multifunctional coupling coordination degree of cultivated land between counties and districts in Hebei Province and used the global Moran’s I to measure the global autocorrelation index [35]. The formula is as follows [35]:

$$I=\frac{\sum_{i=1}^{n}\sum_{j=1}^{n}w_{ij}(x_{i}-\bar{x})(x_{j}-\bar{x})}{\frac{1}{n}\sum_{i=1}^{n}{\left(x_{i}-\bar{x}\right)}^{2}\sum_{i=1}^{n}\sum_{j=1}^{n}w_{ij}}$$ (25)

where *w*_*ij*_ is the spatial weight of county *i* and county *j*, and *n* is the number of counties. The range of *Moran’s I* is [–1,1]. If *Moran’s I* is less than 0, there is a negative spatial correlation, which indicates agglomeration of objects with different attributes. If *Moran’s I* is 0, there is no spatial correlation, which indicates a random distribution. If *Moran’s I* is greater than 0, there is a positive spatial correlation, which indicates agglomeration of objects with similar attributes. The larger the value of *Moran’s I* is, the more obvious the spatial aggregation effect is.

*(2) Local autocorrelation analysis*. Global autocorrelation can represent the average aggregation or dispersion of similar attributes, but it cannot accurately express the spatial agglomeration characteristics of attributes [36]. In this paper, local autocorrelation was introduced to reveal the degree of correlation of similar attributes in the local space of the research unit. The local Moran’s index can reflect the degree of correlation between the multiple functions of cultivated land in each county and the adjacent cities. The formula is as follows [36]:

$$I_{i}=\frac{\left(x_{i}-\bar{x}\right)}{\frac{1}{n}\sum_{i=1}^{n}{\left(x_{i}-\bar{x}\right)}^{2}}\sum_{j=1}^{n}w_{ij}\left(x_{j}-\bar{x}\right)$$ (26)

where *I*_*i*_ is the local *Moran’s I* index, *w*_*ij*_ is the spatial weight matrix, and *x*_*i*_ and *x*_*j*_ are the coupling coordination values of the *ith* and *jth* research units, respectively.

## Results

### Multifunctional temporal and spatial variation characteristics of cultivated land

#### Multifunctional temporal variation characteristics of cultivated land

**Fig 2:**
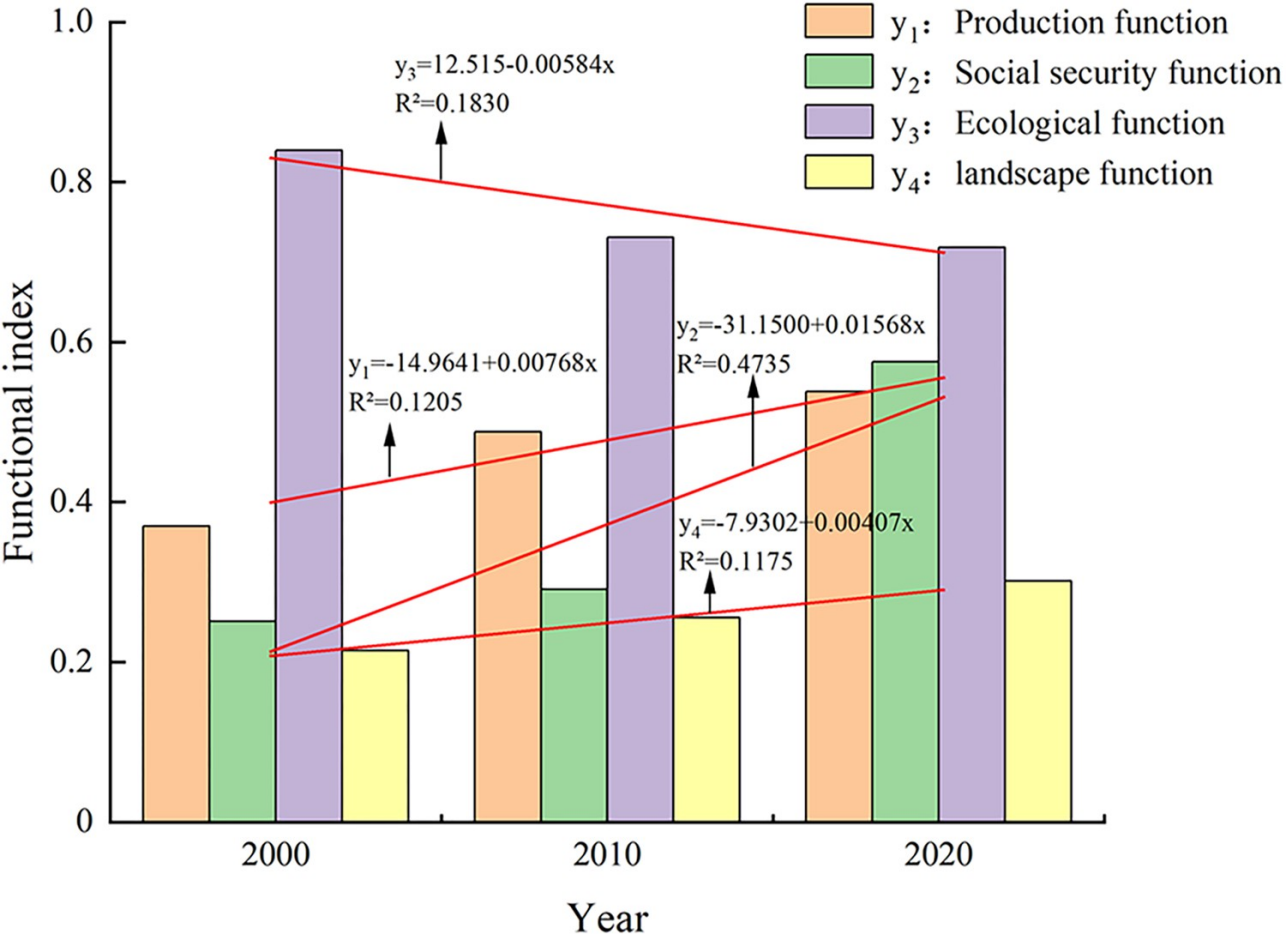
Multifunctional index of cultivated land in Hebei Province from 2000 to 2020.

Based on the calculation formula of the function index, the production function, social function, ecological function and landscape function index of cultivated land in Hebei Province from 2000 to 2020 were calculated, and the time series changes were measured and analyzed. The temporal variation in cultivated land function is shown in Fig 2. According to the trendline function, it can be inferred that from 2000 to 2020, except for the ecological function, which exhibited a declining trend, the trendline slopes (*k* values) of the other functions, including production, social security, and landscape, were all greater than 0, indicating an upward trend. The upward trend in the social security function was particularly prominent, with an R^2^ value exceeding 0.4. The ecological function index decreased from 0.8396 in 2000 to 0.7186 in 2020. Although there was a downward trend, the change amplitude was small, and its value was always greater than that of the other functions; furthermore, it maintained its functional grade at a strong level or above. The production function index generally showed a steady upward trend. The function index increased from 0.3699 in 2000 to 0.5384 in 2020, with a difference of 0.1685. The function level increased from a weak level to a medium level. The *K* value of the social security function was the largest, the change range was the largest, and the functional level increased from a weak level to a medium level, with an extreme value of 0.3246. The landscape function index showed a continuous increasing trend, increasing from 0.2142 in 2000 to 0.3015 in 2020. Its overall growth rate was small; furthermore, its grade did not increase and remained weak. Fig 2 shows that the function of cultivated land in Hebei Province was ranked as ecological function > production function > social security function > landscape function. The gap between each function was significant, especially that between the social security function and the landscape function of cultivated land, which still had room for improvement. Although the comprehensive function of cultivated land increased from 0.3380 in 2000 to 0.5939 in 2020, it was close to the higher functional level.

#### Multifunctional spatial variation characteristics of cultivated land

**Fig 3:**
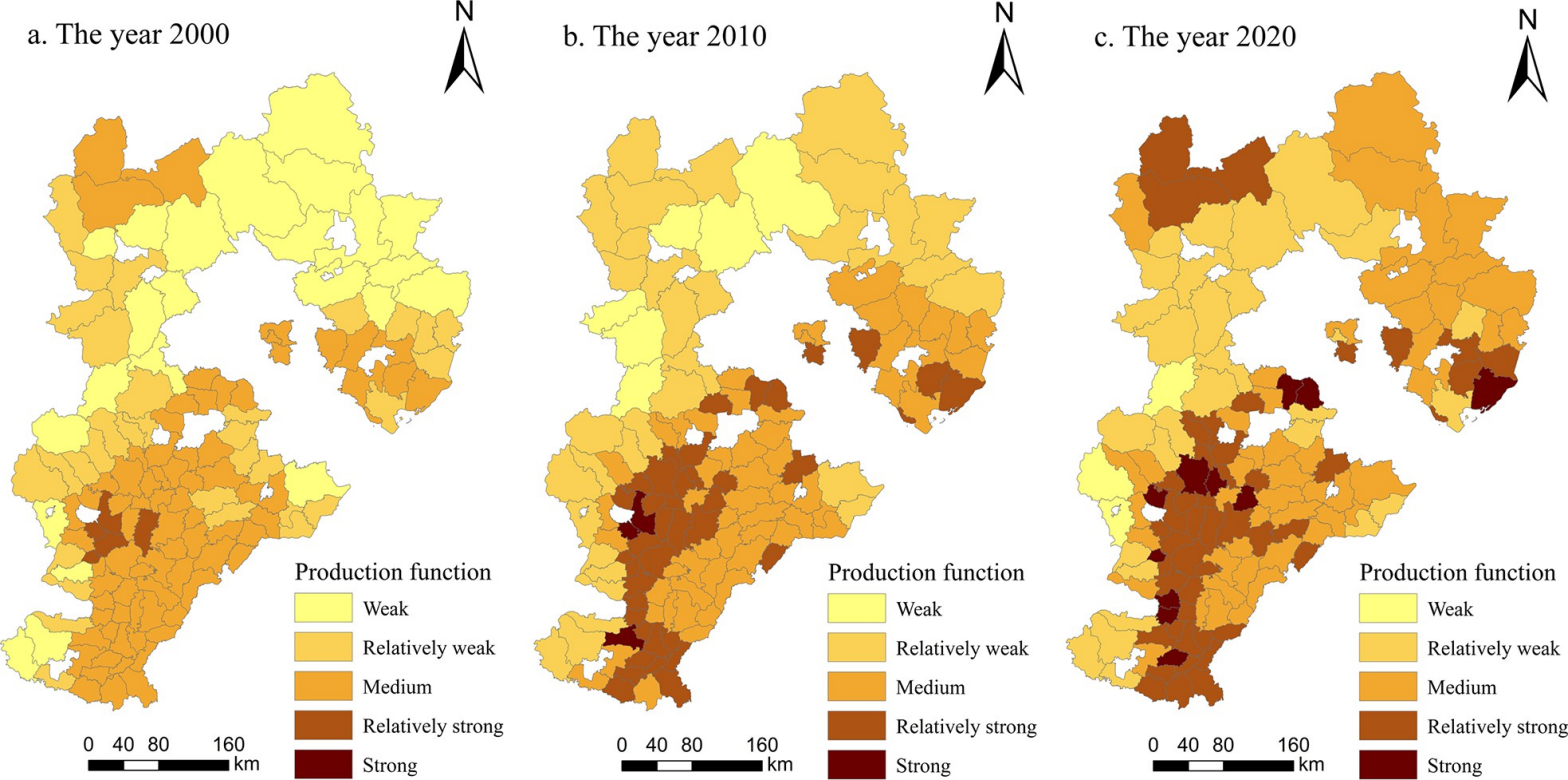
Spatial changes in the production function of cultivated land in Hebei Province from 2000 to 2020 (drawn with ArcGIS).

*(1) Production function*. As shown in Fig 3, from 2000 to 2020, the overall production function of cultivated land in Hebei Province showed a trend of gradual development from weak and medium grades to medium and strong grades. The spatial characteristics were ’high in the south and low in the north’ and had a contiguous agglomeration. The number of weak-level areas decreased from 23 in 2000 to 3 county-level units in 2020, and the number of weak-level counties decreased from 32 to 29. These were mainly distributed in the Taihang Mountains and Yanshan Mountains, while the remaining cities were scattered. The Taihang Mountains and Yanshan Mountains have hilly terrains with limited cultivated land resources. The land reclamation rate is low, resulting in small-scale cultivation that is not suitable for large-scale farming. The production capacity is poor, leading to a weak ability to produce grains. The number of medium-level counties and districts decreased from 75 to 47, which was still the largest number of functional grades. The number of strong grades increased from 5 to 45, second only to the number of medium grades. The production function was mainly based on these two factors, and the plants were widely distributed throughout the study area. In 2000, there were no areas with strong functional grades, while by 2020, there were 11 such areas, all of which were distributed in plain areas. The results showed that the North China Plain in southern Hebei Province is flat and has rich and contiguous cultivated land resources. The production of agricultural products is stable, the production capacity of cultivated land is strong, and its production function is greater than that of the northern mountainous area.

**Fig 4:**
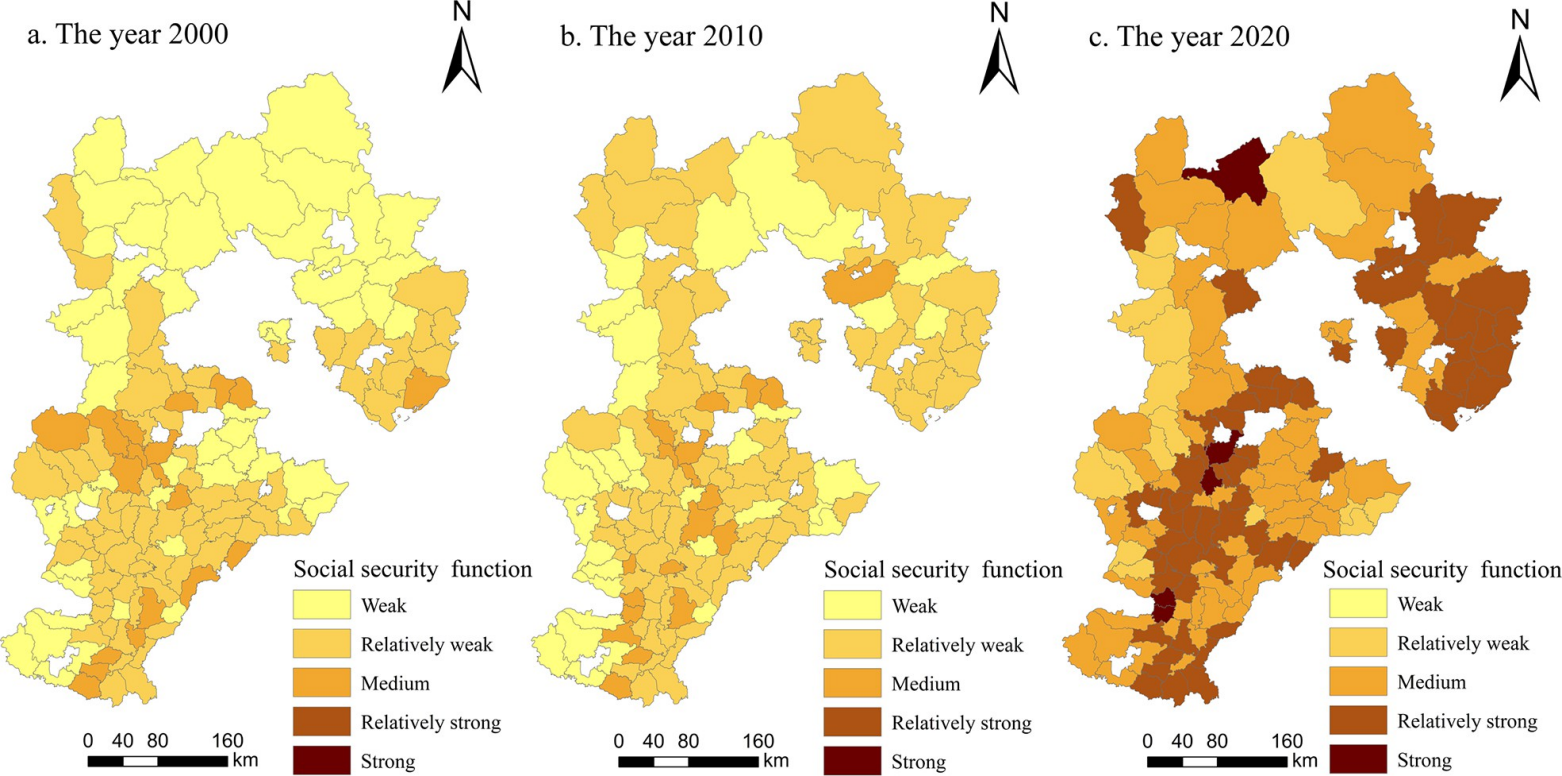
Spatial changes in the social security function of cultivated land in Hebei Province from 2000 to 2020 (drawn with ArcGIS).

*(2) Social security function*. As shown in Fig 4, the social security function of cultivated land in Hebei Province demonstrated a significant growth trend from 2000 to 2020, and the spatial distribution structure was similar to that of the ’+’ shape of ’high in the south and low in the north, high in the east and low in the west’; it was dominated by medium and strong functional grades. In 2000, the social security functions of 116 counties and districts were at weak and relatively weak levels, and only 19 counties and districts were at medium levels. From 2000 to 2010, the social security function of the study area increased but not significantly, and the total level numbers did not change. Only 15 counties and districts improved from a weak functional level to a relatively weak functional level. From 2010 to 2020, there was significant growth in the social security function within the research area, with a total of 119 counties achieving medium or higher levels. This led to the emergence of counties classified under the strong and very strong functional levels. On the premise of fully developing and utilizing cultivated land resources, farmers in Guyuan County, Renze District, Nanhe District, Qingyuan District and Anguo City continuously improved the production capacity of grain-suitable cultivated land, increasing the social security function of cultivated land in these areas to strong levels. Additionally, some farmers tended to plant economic crops with greater economic benefits to adapt to changes in comparative advantages. Therefore, the social security function of cultivated land appeared in these areas. During the study period, the social security function value of Hebei Province showed a certain degree of spatial agglomeration.

**Fig 5:**
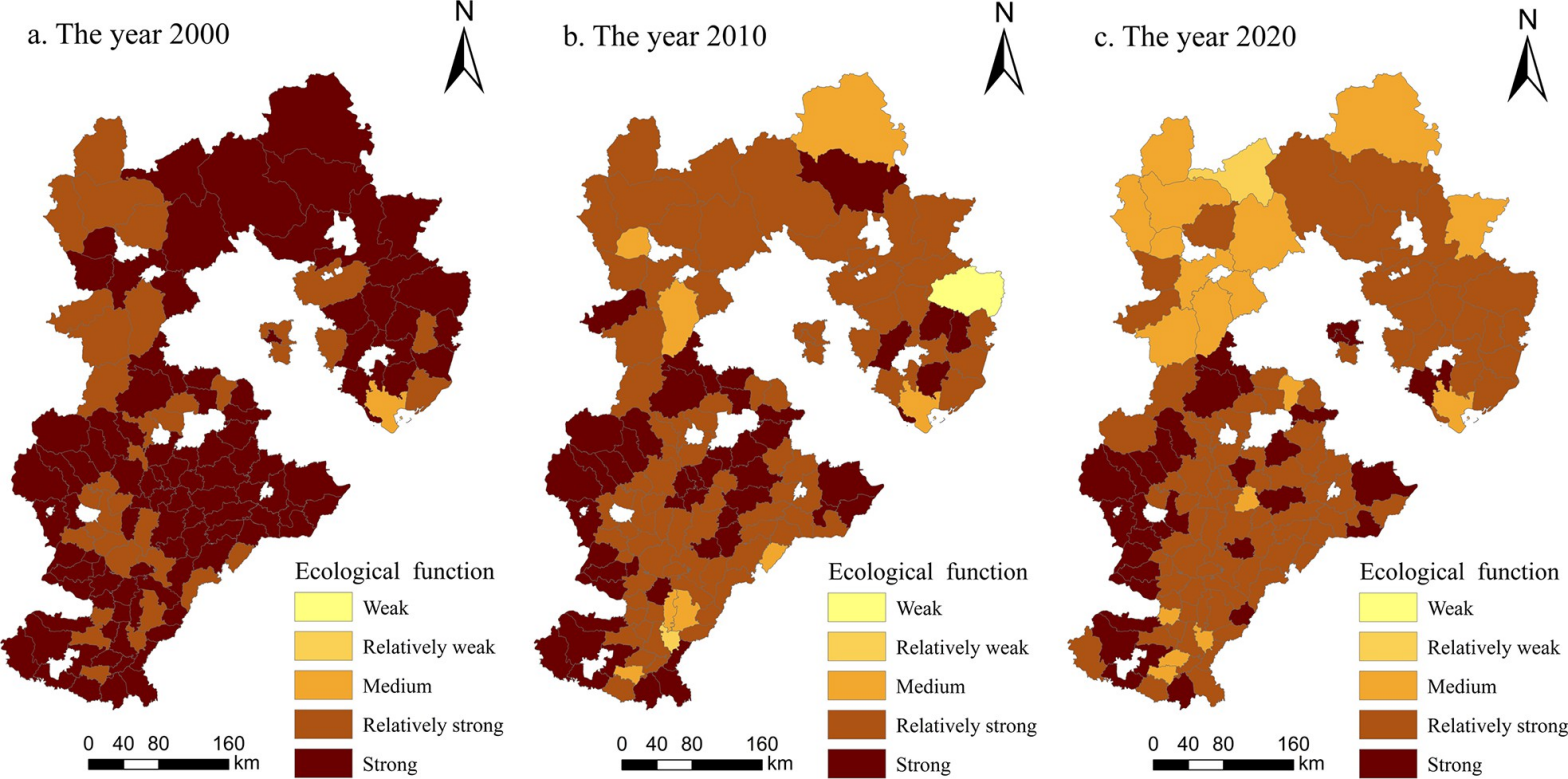
Spatial changes in the ecological functions of cultivated land in Hebei Province from 2000 to 2020 (drawn with ArcGIS).

*(3) Ecological function*. As shown in Fig 5, from 2000 to 2020, the ecological function of cultivated land in Hebei Province generally showed a slight downward trend, and the spatial distribution was roughly characterized by ’high in the south and low in the north’. The overall level was dominated by relatively strong and strong functional grades, and the proportion decreased from 100% to 86%. Among them, 64 county-level units decreased from strong grades to relatively strong grades, and 3 county-level units increased from relatively strong grades to strong grades. The reason is that grain crops are most commonly cultivated in Hebei Province; moreover, agriculture has gradually achieved centralized utilization and scale development, the planting structure tends to be consistent, and biodiversity has decreased. At the same time, the economic growth of Hebei Province has intensified farmers’ dissatisfaction with agricultural income. To increase production, they need to invest more resources, such as chemical fertilizers, pesticides, and machinery. These two factors led to a slight decline in the ecological function of cultivated land. During the study period, counties with relatively strong and strong grades appeared in the North China Plain and mountainous and hilly areas and showed spatial aggregation characteristics of ecological environmental functions. Specifically, the farmland ecosystem in the North China Plain has rich and diverse biodiversity, and the per capita ecological carrying capacity of cultivated land is high, so cultivated land has many ecological functions. In contrast, the topography of the Taihang Mountains and Yanshan hilly areas is undulating, which is not suitable for large-scale farming, resulting in less use of chemical fertilizers, pesticides and mulch. Therefore, the ecological environment of cultivated land is less affected by humans in these areas, and the natural conditions are better.

**Fig 6:**
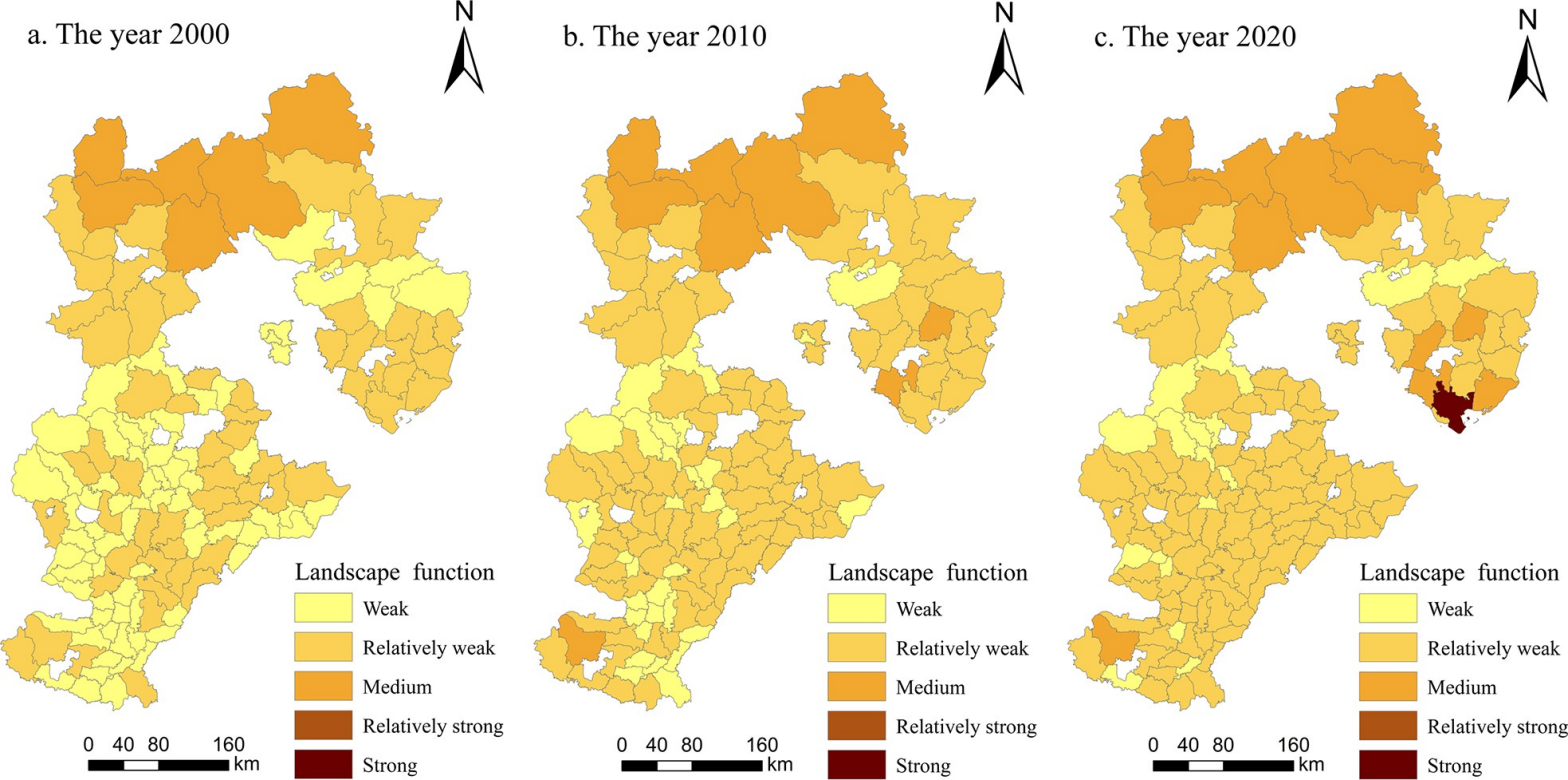
Spatial changes in the landscape function of cultivated land in Hebei Province from 2000 to 2020 (drawn with ArcGIS).

*(4) Landscape function*. As shown in Fig 6, the landscape function of cultivated land in Hebei Province demonstrated an overall upward trend from 2000 to 2020, the spatial distribution was generally characterized by ’low in the south and high in the north’, and its overall function was relatively weak. In 2000, among the 135 county-level units in the study area, the number of counties with relatively weak levels accounted for 53%, and only 6 counties were at medium levels, namely, Fengning County, Weichang County, Chicheng County, Guyuan County, Kangbao County, and Zhangbei County, with no units classified as having relatively strong or strong functional levels. In 2010, the landscape function was dominated by weak grades and accounted for 70%. Compared with 2000, the number of medium-grade counties increased by 3. From 2010 to 2020, 21 county-level units changed, of which only Zanhuang County and Cixian County decreased in function, and the remaining 19 counties increased their functions. At the end of the study, the Caofeidian District of Tangshan city was the only place that was rated as a strong level area. It is located in the two core areas of the Bohai Rim and the Beijing-Tianjin Rim. Its ecotourism environment is unique, with the second largest state-owned farm in the country, and it has outstanding water agricultural characteristics. In addition, farmland landscape ecosystems in this area have a certain scale.

#### Analysis of the multifunctional coupling coordination degree of cultivated land

**Fig 7:**
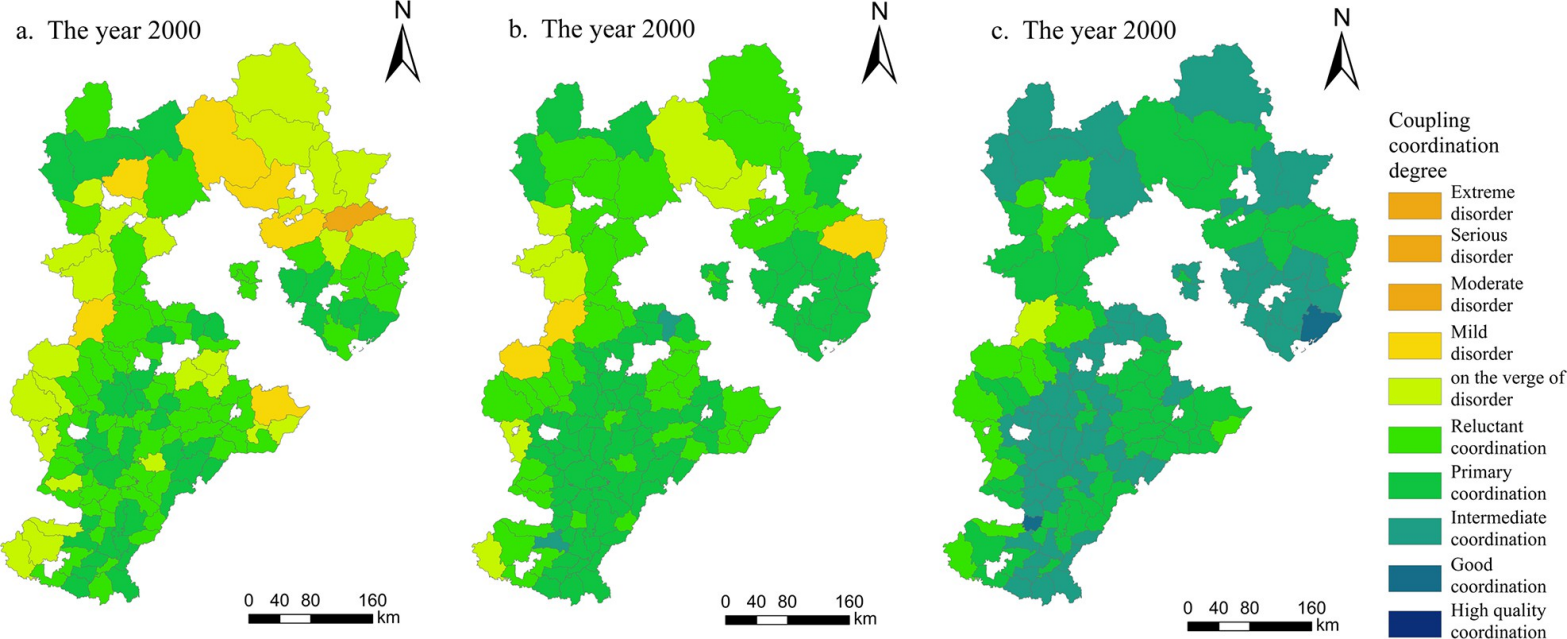
Spatial distribution of the multifunctional coupling coordination degree of cultivated land in Hebei Province from 2000 to 2020 (drawn with ArcGIS).

As shown in Fig 7, the multifunctional coupling coordination degree of cultivated land in Hebei Province showed a significant upward trend from 2000 to 2020, and the coupling coordination type was mainly coordination. It changed from "moderate imbalance-primary coordination" in 2000 to "on the verge of imbalance-good coordination" in 2020, improving by two levels. There were no units classified at the three levels of extreme imbalance, serious imbalance and high-quality coordination during the study period. In 2000, 2010 and 2020, the proportions of counties with coupling coordination degrees greater than 0.5 and in a coordinated state were 77.03, 92.59 and 99.26, respectively, showing the characteristics of a large quantity, a wide distribution and an obvious agglomeration trend. During the period from 2000 to 2020, Hebei Province accelerated the process of urbanization and the rapid development of high-end industries and modern agriculture, leading to an increase in the number of agricultural workers, a greater capacity of the rural labor force, and an increase in net income from household farming. Thus, the province enhanced the social security function of cultivated land. Moreover, the large market demand brought about by urban development stimulated the substantial growth of grain output and agricultural output value and further enhanced the production function of cultivated land in the county. However, the scale of agricultural management led to a decline in the diversity index of agricultural land, and the excessive use of fertilizers, pesticides and mulch weakened the ecological function of cultivated land. Nevertheless, the transformation of cultivated land and the development of modern agriculture enhanced the contiguity of cultivated land, increased the per capita GDP, and improved the landscape function of cultivated land.

In 2000, the multifunctional coupling coordination degree of cultivated land in Hebei Province mainly exhibited limited coordination and accounted for 46.67%. Kuancheng County in Chengde city was moderately imbalanced, and its coupling coordination degree was the lowest. The city’s production and landscape function indices were the lowest, and its social security function was at the weak function level. The 6 counties of Laiyuan County, Huanghua city, Fengning County, Luanping County, Xinglong County and Chongli District were classified in the mild imbalance stage, and 24 counties were on the verge of imbalance; these counties were distributed mainly in the Taihang Mountains and Yanshan Mountains. The results showed that the cultivated land production and social security index in each dysfunctional area of cultivated land were low, which was quite different from the ecological function index. Compared with 2000, the multifunctional coupling coordination degree of cultivated land in Hebei Province increased in 2010 and changed from reluctant coordination to primary coordination, accounting for 59.26%. The lowest levels, found in Fuping County, Laiyuan County of Baoding city and Qinglong County of Qinhuangdao city, were mild imbalance while the coupling coordination indices were 0.377, 0.361 and 0.387, respectively. The number of counties on the verge of imbalance decreased to 7; these counties were still distributed in the Taihang Mountains and Yanshan Mountains. The number of primary coordinators increased from 41 to 80; these coordinators were mainly distributed in southern Hebei Province and were scattered in northwestern and eastern Hebei Province. There were two areas with intermediate coordination, namely, Yongnian District of Handan city and Gu’an County of Langfang city. These two counties had greater production functions, social security and ecological function indices and greater degrees of coupling coordination. In 2020, the multifunctional coupling coordination degree of cultivated land in Hebei Province continued to increase, and the whole province was in a coordinated development state except for Laiyuan County. Laiyuan County is in a deep mountain area at the northern end of the Taihang Mountains. Its reclamation rate of cultivated land was low, and its cultivated land was not suitable for large-scale operations. These circumstances led to less food production and lower development and utilization. This made the production function and social security function indices of cultivated land smaller and in a state of near imbalance. Thirteen counties with barely coordinated development were concentrated in the western part of Hebei Province with weak production functions, while 54 counties with primary coordinated development were distributed in all cities of Hebei Province. The counties with intermediate coordinated development were roughly distributed in a ’T’ shape, increasing from 2 in 2010 to 65. The newly added counties with good coordinated development were Laoting County of Tangshan city and Nanhe District of Xingtai city, which were the two counties with the highest coupling coordination degrees; their coupling coordination degree values were 0.806 and 0.817, respectively.

#### Spatial autocorrelation analysis of the multifunctional coupling coordination degree of cultivated land

**Fig 8:**
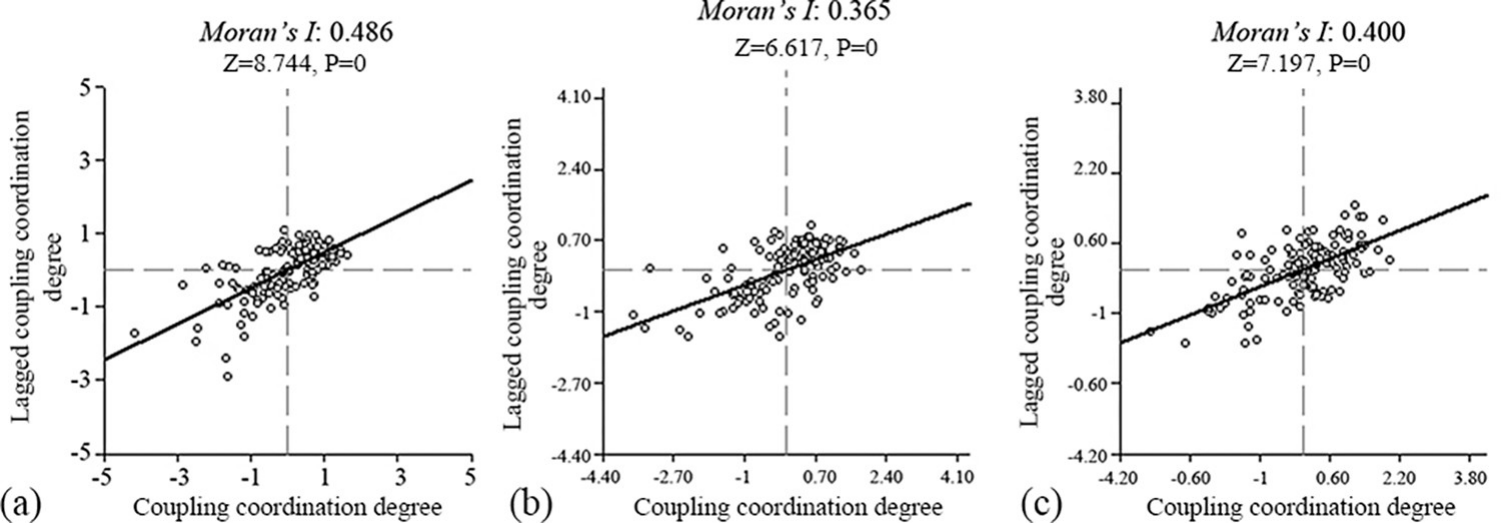
Moran’s index scatter map of the multifunctional coupling coordination degree of cultivated land in Hebei Province from 2000 to 2020. (a) The year of 2000. (b) The year of 2010. (c) The year of 2020.

To explore the spatial patterns and changes in the multifunctional coupling coordination degree of cultivated land in Hebei Province, GeoDa software was used to analyze the global spatial autocorrelation of the coupling coordination degree to obtain the Moran’s index of each function and create a Moran’s index scatter diagram (Fig 8). From 2000 to 2020, the Moran’s indices of the multifunctional coupling coordination degree of cultivated land in Hebei Province were 0.486, 0.365 and 0.400, all of which were greater than 0 (objects with similar attributes cluster). This result indicated that the coupling coordination degree of Hebei Province had a positive spatial correlation during the study period; that is, the counties and districts of Hebei Province did not develop in isolation. Although the Moran’s I value displayed a ’V’-type change (a decease followed by an increase) during the study period, the fluctuation was small, which indicates that this positive spatial correlation was relatively stable. Furthermore, the Z values were 8.744, 6.617 and 7.197, respectively (all were greater than 2.58), and all P values were <0.01, which indicate significant spatial correlation among the counties and districts and that the coupling coordination degree of each county/district was significantly influenced by its neighbors. The scatter plot shows that the points of the multifunctional coupling coordination degree of cultivated land in the study area were mainly concentrated in the first quadrant (high-high (H-H) agglomeration) and the third quadrant (low-low (L-L) agglomeration), while the second quadrant (low-high (L-H) agglomeration) and the fourth quadrant (high-low (H-L) agglomeration) had fewer points, which indicate relatively high spatial positive correlation and agglomeration of the multifunctional coupling coordination degree of the cultivated land in the investigated province. These findings suggest that all counties and districts in Hebei Province should accelerate the realization of win-win cooperation with surrounding areas and promote a higher level of multifunctional coupling coordination of the cultivated land in the future.

**Fig 9:**
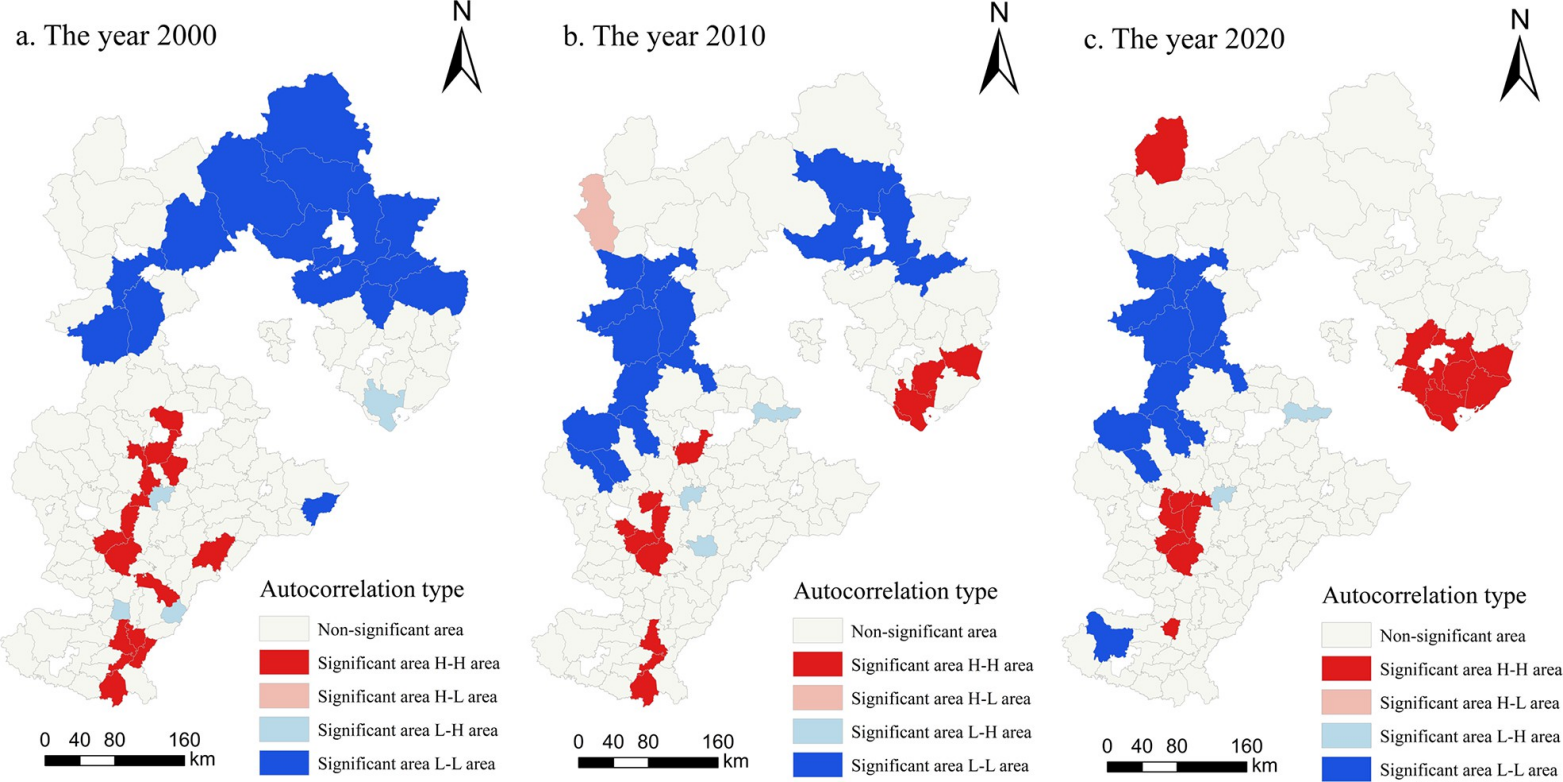
Local spatial correlation diagram of the multifunctional coupling coordination degree of cultivated land in Hebei Province from 2000 to 2020 (drawn with ArcGIS).

To further explore the multifunctional coupling and coordination relationship of cultivated land and its spatial pattern distribution changes, the local spatial autocorrelation analysis model was used to obtain the local spatial correlation map of cultivated land function. Fig 9 shows the spatial distribution and agglomeration characteristics of the multifunctional coupling and coordination level in Hebei Province. In Fig 9, the functional coupling coordination level of each county and its spatial agglomeration characteristics with other surrounding counties are represented by different colors. From 2000 to 2020, the counties in Hebei Province with multifunctional coupling coordination levels of cultivated land located in significant H-H areas shifted from a centralized distribution in the southern part of Hebei Province to the east and presented a triangular three-point distribution. The counties with a significant L-L distribution shifted from the northern part of Hebei Province to the western part. The counties with a significant H-L distribution did not exist in 2000 or 2020, and only Shangyi County was included in 2010. The number of counties with a significant L-H distribution gradually decreased to two; these counties were Bazhou city and Anping County. From a distribution of agglomeration perspective, the overall distribution of cities with various types of agglomeration characteristics was relatively stable. Among them, the number of cities with the H-H agglomeration type was the highest, followed by those with the L-L agglomeration type; there were slight changes in the H-L agglomeration type and L-H agglomeration type. The number of counties with H-H agglomeration types decreased from 16 to 15. The county-level units in this area had a high coordination effect, and the development of various functions of cultivated land was relatively balanced; thus, the coupling coordination degree of cultivated land was high. The number of cities with L-L agglomeration types decreased from 15 to 10 counties. The cultivated land production, social security and landscape functions in this area were low, and the coordination degrees between various cultivated land functions were low, which led to low coordination radiation between counties. Shangyi County was the main area with a H-L agglomeration distribution, which showed that it had greater urban coupling coordination than did the surrounding cities. The coupling coordination levels of cities with L-H agglomeration types were lower than those of surrounding cities. For other cities without obvious agglomeration characteristics, correlations with surrounding cities and other cities were weak; these areas were in an isolated state of development and did not play a role in promoting each other. In general, the spatial distribution of the actual coupling coordination degree was basically consistent with the spatial correlation diagram obtained by local spatial autocorrelation analysis.

## Discussion

In this study, we investigated the spatiotemporal changes in the multifunctionality and coupling coordination degree of the county-level cultivated land in Hebei Province, and in the meantime, we analyzed the agglomeration status of the multifunctional coupling coordination degree of the cultivated land in Hebei Province, in combination with spatial autocorrelation analysis. The findings of this study may provide reference for different regions in Hebei Province to take advantage of cultivated land according to different functions and thus to realize the optimization of the comprehensive benefits of cultivated land utilization. Furthermore, this study may also provide scientific basis and theoretical support for the sustainable utilization and management of cultivated land resources in regions with similar human and geographical environments across the world.

In this study, we analyzed the time series changes in the production, social, ecological and landscape functions of the cultivated land in Hebei from 2000 to 2020 based on the functional index formula, and the results showed that all functions displayed increasing trends during the investigated years, except for the ecological function, which exhibited a decreasing trend (Fig 2). Among the various functions, the social security function exhibited the largest variation, whose extreme value was 0.3246. Liu et al. [35] calculated the functional values of cultivated land in China by using the value method and then analyzed the spatiotemporal evolution characteristics of multifunctional cultivated land in China from 2010 to 2020. They found that the comprehensive functional value of cultivated land in China rose, as ecological, social security, and landscape functions all exhibited an increase; however, the production function initially decreased before increasing. Our results are basically consistent with their results. Based on the results of the multifunctional temporal changes in farmland, attention should be given to landscape functions in the future. When transforming farmland, the regularity and diversity of farmland should be improved and increases in and balanced distributions of landscape types should be promoted. When improving the production function of farmland, attention should also be given to the use of fertilizers and other materials to avoid reducing the ecological function of farmland.

Li et al. [23] calculated the functional values of the Two Lakes Plain by using the weighted sum method and analyzed the spatiotemporal evolution characteristics and trade-off synergies of farmland multifunctionality. They found that farmland multifunctionality presented noticeable regional spatial characteristics. Our results also showed regional differences in cultivated land multifunctionality of Hebei Province, that is, the overall production function had a distributional characteristic of being high in the south and low in the north (Fig 3), the social security function was high in the south and east and low in the north and west (Fig 4), the ecological function was characterized by being high in the south and low in the north (Fig 5) and the landscape function demonstrated a distribution of being low in the south and high in the north (Fig 6). The spatial distribution differences of arable land resources in Hebei Province are influenced by terrain and topography. The overall multifunctional values of arable land in mountainous cities are relatively weak, while the multifunctional values of arable land in plain areas are relatively strong. According to the findings of this study, the governments in each county and district should re-examine the various functions of cultivated land; based on changing trends, they can combine regional characteristics with the advantages of cultivated land resources to rationally utilize and enhance the value of cultivated land, thus providing better services that help protect cultivated land.

Our study showed that the multifunctional coupling coordination of cultivated land in Hebei Province exhibited noticeable temporal and spatial changes between 2000 and 2020 (Fig 7). Zhang et al. [36] took the provinces in the Yellow River basin to investigate the spatiotemporal evolution characteristics and driving factors of the coupling coordination level of the “production-life-ecology” function in the Yellow River Basin via a coupling coordination degree model. They found that the overall level of coupling coordination improved compared with that in the past, and the gap in coupling coordination between provinces continued to narrow; this finding is consistent with our findings. However, they divided the coupling coordination level into five stages with a 0.2 interval based on the province as the research unit. In this study, the degree of coupling coordination was divided into ten stages and two categories, with a 0.1 interval as the research unit for each county. As a consequence, five additional stages were added to the classification; thus, this more refined classification should result in more accurate results. In the future, each county should propose suitable paths for the development of arable land based on the spatial differences and evolution laws of coupling coordination levels and promote the sustainable utilization of arable land resources. At the same time, great importance should be attached to the radiation driving ability of regions with high coupling and coordination, and support for low value regions should be increased.

The multifunctional coupling coordination of cultivated land in Hebei Province also showed strong spatial positive correlation and spatial concentration, exhibiting bipolar agglomeration characteristics of H-H and L-L (Fig 9). Wang et al. [37] investigated the spatiotemporal characteristics and coupling coordination of land functions in the Yangtze River urban agglomeration from 2005 to 2020 and analyzed the spatial clustering of coupling coordination effects in urban agglomerations based on the coupling coordination model, kernel density curves, and the spatial autocorrelation model. They also found a significant spatial positive correlation in the coupling and coordination between various functions, with a certain clustering phenomenon in high- and low-value areas. However, their spatial autocorrelation analysis focused only on global spatial autocorrelation analysis, and their analysis considered only the temporal dimension. In contrast, we included local spatial autocorrelation analysis in the current study; therefore, the spatial distribution and clustering characteristics of the coupling coordination levels of the investigated region could also be analyzed. According to local autocorrelation, H-H-type counties and districts in Hebei Province exhibited a shift in concentration from the southern region to the eastern region, while L-L-type counties and districts showed a shift in concentration from the northern region to the western region. Based on these findings, Hebei Province should follow the regional coordinated development model, focus on spatial agglomeration advantages, and utilize functional advantages to drive the development of functionally disadvantaged areas.

Based on the findings with regard to the functions and coupling coordination of cultivated land in the counties and districts of Hebei Province, the following suggestions can be proposed to promote the coordinated multifunctional development of the cultivated land. For counties and districts with coordinated multifunctional development, such as Leting County and Nanhe District, industries that demonstrate the diverse value of cultivated land can be created to construct a modern rural industrial system, and the deep processing industry of agricultural products can be strengthened. For districts and counties that focus on production function, such as Dingzhou City, Gu’an County and Raoyang County, while maintaining this function as the basis of development, it is plausible to emphasize the ecological value of cultivated land, improve the internal ecological environment of cultivated land, consolidate the foundation of agricultural product production and implement the strategy of storing grain based on technological application to continuously strengthen the production capacity. For districts and counties with social function as the main focus, such as Anguo City and Guyuan County, production function should be improved and importance to ecological functions should be attached. With the market as the guide, the internal structure of the crop industry should be further adjusted and optimized and the ecological environment of the cultivated soil should be improved to promote the sustained development of green agriculture. For those with ecological function as the main focus, such as Yixian, Pingshan County and Wu’an City, ecological function can be considered as the foundation with landscape function as the highlight to exert the demonstration role of green agricultural product production bases, improve the comprehensive crop production level and enhance the benefits of agriculture and tourism integration development. For landscape function-focused counties and districts, such as Caofeidian District, Longhua County and Zhangbei County, it is plausible to take landscape function as the lead with ecological function as the combination point to develop ecological tourism agriculture and thus to promote the multifunctional utilization of the cultivated land.

### Conclusion

In this study, a targeted index system was constructed using production, social security, ecology and landscape functions as the main functions of cultivated land. Based on this system, the multifunctionality of cultivated land at the county level in Hebei Province was evaluated. To achieve this goal, the CRITIC method was combined with the entropy weight method to determine the index weight. The spatiotemporal changes in multifunctional cultivated land in Hebei Province in 2000, 2010, and 2020 were analyzed using a linear regression model and ArcGIS software, and the spatiotemporal characteristics and agglomeration status of the multifunctional coupling coordination degree of cultivated land were further explored using a coupling coordination degree model and spatial autocorrelation analysis. This study led to the following main findings.

1. From 2000 to 2020, the production, social security and landscape function of cultivated land in Hebei Province showed an overall upward trend, and the ecological function showed a slight downward trend. From the perspective of spatial distribution, the high-level areas of cultivated land production function in Hebei Province were distributed mainly in the North China Plain in the south, while the low-level areas were distributed mainly in the Taihang Mountains and Yanshan Mountains. The social security function of cultivated land showed a distribution structure similar to a ’**+**’ shape (i.e., high in the south and low in the north, high in the east and low in the west), and the counties with medium and relatively strong functional grades were dominant. Counties with relatively strong and strong grades for the ecological function of cultivated land were concentrated in the North China Plain and mountainous hilly areas. The overall landscape function of cultivated land was dominated by relatively weak functional grades, which were mainly distributed in Zhangjiakou city and Chengde city, and exhibited the distribution characteristics of ’low in the south and high in the north’.
2. From 2000 to 2020, the multifunctional coupling coordination degree of cultivated land in Hebei Province showed a significant upward trend in general and changed from limited coordination to intermediate coordination. At the end of the study period, all provinces except Laiyuan County were in a state of coordinated development. The areas with high coupling coordination were distributed mainly in southern Hebei Province. In contrast, the areas with low coupling coordination were concentrated mainly in the Taihang Mountains and Yanshan Mountains. To realize the balanced and coordinated development of each function, each region should protect cultivated land resources according to its own conditions and strengthen the connections between each function.
3. According to the global autocorrelation results, the Moran’s index of the multifunctional coupling coordination degree of cultivated land in Hebei Province from 2000 to 2020 was positive, which indicated that the coupling coordination degree of Hebei Province during the study period exhibited a positive spatial correlation. The spatial distribution had a certain regularity, and the spatial correlation showed a ’V’-type trend of first decreasing and then increasing. According to the local autocorrelation results, most of the districts and counties exhibited H-H agglomeration or L-L agglomeration. This finding also showed that the multifunctional coordination of cultivated land in Hebei Province presented the characteristics of H-H and L-L agglomerations and was basically consistent with the actual situation of its spatial distribution. The H-H-type counties were distributed in the southern part of Hebei Province and shifted to the eastern part, forming a triangular three-point distribution pattern. The distribution of L-L counties was concentrated in the northern part of Hebei Province and shifted to the western part.

Research on the multifunctional coupling coordination degree of cultivated land plays an important role in clarifying the coordinated development and agglomeration characteristics of cultivated land production, social security, ecology and landscape functions. By considering the natural conditions of different regions and the development needs of people in different periods, a suitable development path for cultivated land can be proposed to provide a theoretical reference for the healthy and coordinated development of cultivated land, thus promoting the sustainable use of cultivated land resources. The findings of this study might provide a scientific basis and theoretical support for the sustainable utilization and management of cultivated land resources in areas with similar human and geographical environments and realize the optimization of the comprehensive benefits of cultivated land use.

However, this study has some limitations, and further studies should be conducted in the future. First, data sources for analysis need to be expanded. Considering availability, only social statistics and land use status data were included in this study, while those concerning elevation, soil, and meteorology were comparatively insufficient, which led to certain limitations in the selection of indicators and the construction of the evaluation indicator system. Therefore, data sources can be expanded, and evaluation indicators can be enriched to verify the outcomes of this study. Second, the evaluation index system needs to be improved. Due to the lack of unified classification of farmland functions, we selected four major functions for evaluation in this study based on the literature as well as the development of the concept of the multifunctionality of cultivated land in China. However, with the development of China’s economy and society and the increasing diversity of people’s lives, functional diversity has gradually gained the favor of scholars. In the future, the types of evaluation functions could be considered more fully to make the evaluation index system more comprehensive. Finally, the research scale was not small enough, and there has been no multiyear, continuous analysis or evaluation of the multifunctional development status of cultivated land. In the future, the grid can be considered the minimum evaluation unit for evaluating the evolution law of multifunctional farmland from a more micro- and detailed perspective, and continuous annual multifunctional evaluations of farmland can be performed to improve the precision of suggestions for the multifunctional protection of farmland.
